# Radiation-Induced Remodeling of Bone Metastases and the Bone Marrow Microenvironment: From External-Beam Radiotherapy to Radiopharmaceutical Therapy

**DOI:** 10.3390/cancers18213500

**Published:** 2026-10-30

**Authors:** Satoru Monzen, Akihito Kotani, Masaki Tatara, Atsushi Muro, Yasuyo Fukushi, Andrzej Wojcik, Yasushi Mariya

**Affiliations:** 1 Department of Radiation Science, Graduate School of Health Sciences, Hirosaki University, Hirosaki 036-8564, Japan; 2 Research Center for Biomedical Sciences, Hirosaki University Graduate School of Health Sciences, Hirosaki 036-8564, Japan; 3 Department of Nursing, Faculty of Medical Technology, Teikyo University, Tokyo 173-8605, Japan; 4 Department of Molecular Biosciences, The Wenner-Gren Institute, Stockholm University, SE-10691 Stockholm, Sweden; andrzej.wojcik@su.se; 5 Department of Radiology, Aomori Rosai Hospital, Hachinohe 031-8551, Japan; mariya@hirosaki-u.ac.jp

**Keywords:** bone metastasis, bone marrow microenvironment, radiotherapy, radiopharmaceutical therapy, hematopoietic stem cells, oxidative stress, bone remodeling, multi-omics, metabolomics, lipidomics

## Abstract

**Background/Objectives:** Bone metastases arise within a specialized microenvironment in which tumor cells interact with skeletal, stromal, vascular, immune, and hematopoietic compartments. Radiotherapy therefore affects not only metastatic tumor cells but also the surrounding bone and marrow niches. This review examines radiation-induced remodeling of the bone metastatic and bone marrow microenvironments across external-beam radiotherapy (EBRT) and radiopharmaceutical therapy (RPT). **Methods:** We performed a narrative synthesis of experimental and clinical studies addressing cellular radiosensitivity, skeletal and stromal remodeling, hematopoietic injury, vascular and immune responses, oxidative and metabolic stress, and spatial and temporal patterns of radiation delivery. Emerging transcriptomic, proteomic, metabolomic, lipidomic, single-cell, and spatial approaches were also considered. **Results:** EBRT and RPT produce overlapping but biologically distinct perturbations because of differences in dose rate, exposure duration, particle range, target localization, microscopic energy deposition, and spatial dose heterogeneity. These differences can influence tumor control, resistance, bone remodeling, and marrow toxicity through effects on osteoblasts, osteoclasts, stromal and vascular cells, immune populations, and HSPCs. These issues are particularly relevant to metastatic castration-resistant prostate cancers, in which bone seeking and tumor targeting radiopharmaceuticals differentially irradiate cellular compartments within the same skeletal environment. **Conclusions:** Bone metastases should be viewed as part of a dynamic bone–tumor–marrow microenvironment. Integrating radiation biology, spatial dosimetry, and multi-omics may support more individualized treatment strategies that maximize tumor control while limiting skeletal and hematologic injury.

## Simple Summary

Bone metastases develop within a complex environment composed not only of tumor cells but also of bone-forming and bone-resorbing cells, stromal cells, immune cells, blood vessels, and hematopoietic cells. Radiotherapy can therefore influence both metastatic tumors and the surrounding bone marrow microenvironment. These effects may differ between external-beam radiotherapy and radiopharmaceutical therapy because of differences in spatial distribution of dose, dose rate and radiation quality. This review summarizes how radiation reshapes the bone–tumor–marrow environment, including changes in bone remodeling, hematopoiesis, oxidative stress, immune responses, and metabolism. It also discusses how transcriptomic, proteomic, metabolomic, lipidomic, and spatial multi-omics approaches may help characterize these changes. A better understanding of these interactions may support treatment strategies that improve tumor control while reducing damage to normal bone marrow.

## 1. Introduction

Bone metastasis is a major manifestation of advanced malignancy, particularly in prostate and breast cancers, and is associated with substantial effects on skeletal integrity, pain, mobility, quality of life, and clinical outcome [1,2,3,4]. The development of skeletal metastases is not simply the consequence of tumor cells reaching mineralized tissue. Disseminated cancer cells enter a highly organized microenvironment in which osteoblast-lineage cells, osteoclasts, mesenchymal stromal cells, endothelial cells, adipocytes, immune populations, and hematopoietic cells interact through spatially and temporally regulated signals [2,3,4]. These interactions influence tumor-cell homing, dormancy, outgrowth, treatment resistance, and progression to clinically evident skeletal disease. In prostate cancer, these relationships are particularly prominent because bone metastases frequently exhibit an osteoblastic phenotype, although osteoclastic activity and bone resorption remain integral components of the metastatic process [5]. Thus, the biological behavior of a skeletal metastasis cannot be understood from the properties of malignant cells alone.

The bone marrow adds another level of biological complexity. It is the principal site of adult hematopoiesis and contains specialized microenvironments that regulate hematopoietic stem and progenitor cell (HSPC) quiescence, self-renewal, differentiation, trafficking, and regeneration after injury [6,7,8]. These functions are supported by heterogeneous stromal and vascular populations distributed across perivascular, sinusoidal, periarteriolar, and bone-associated regions [6,7,8]. Tumor colonization can disrupt these normal relationships by modifying local signaling networks, altering stromal and vascular functions, and competing with hematopoietic cells for supportive niches. Notably, prostate cancer cells have been shown experimentally to occupy hematopoietic stem cell niches in bone marrow, illustrating the direct biological overlap between metastatic colonization and normal hematopoietic regulation [9]. In this review, we use the term bone–tumor–marrow ecosystem to emphasize that metastatic cancer cells, skeletal cells, hematopoietic cells, immune populations, and their supporting stromal and vascular compartments form an anatomically interconnected and dynamically regulated system rather than independent biological entities.

Radiotherapy represents an important therapeutic intervention within this ecosystem. External-beam radiotherapy (EBRT) is well established for the treatment of symptomatic bone metastases and can be delivered using conventional fractionation, and stereotactic approaches according to the clinical setting [10]. Radiopharmaceutical therapy (RPT) has expanded the therapeutic options for metastatic prostate cancer, in which skeletal involvement is particularly common. Radium-223 (^223^Ra), an alpha-emitting and bone-seeking radiopharmaceutical, improved overall survival in patients with symptomatic metastatic castration-resistant prostate cancer (mCRPC) and bone metastases [11], whereas the beta-emitting PSMA-targeted radioligand ^177^Lu-PSMA-617 improved radiographic progression-free and overall survival in patients with PSMA-positive mCRPC [12]. These approaches irradiate metastatic disease through fundamentally different spatial and temporal patterns. EBRT deposits energy according to an externally planned macroscopic dose distribution, whereas RPT is governed by radiopharmaceutical biodistribution, uptake and retention, radioactive decay, particle range, and time-dependent dose rate [13]. Moreover, a bone-seeking agent such as ^223^Ra and a tumor-targeting radioligand such as ^177^Lu-PSMA-617 can irradiate different cellular and anatomical compartments within the same bone metastatic environment.

The consequences of irradiation extend beyond direct tumor-cell killing. The hematopoietic stem and progenitor compartments are vulnerable to ionizing radiation, and persistent DNA damage, oxidative stress, altered proliferative capacity, and senescence can contribute to acute and long-term impairment of hematopoiesis [14]. Radiation can also perturb non-hematopoietic components that support marrow function. Experimental studies have demonstrated radiation-induced proteomic, oxidative-stress, mitochondrial, and secreted lipid changes in bone marrow stromal and osteoblast-lineage cells, indicating that the supporting niche itself is biologically responsive to irradiation [15]. Within metastatic bone, such effects may occur together with changes in osteoblast–osteoclast balance, vascular integrity, inflammatory signaling, immune composition, redox homeostasis, cellular metabolism, and intercellular communication. These responses can lead to divergent outcomes: some contribute to tumor control, whereas others may favor residual disease, adaptive resistance, abnormal skeletal remodeling, or prolonged marrow dysfunction. Accordingly, tumor response and hematologic toxicity should not necessarily be regarded as independent phenomena; both may arise from radiation-induced perturbation of the same bone–tumor–marrow ecosystem.

The cellular heterogeneity of this ecosystem also presents an important methodological challenge. Single-cell transcriptomic studies have revealed multiple stromal, osteoblastic, vascular, and mesenchymal populations within bone marrow that cannot be adequately represented by conventional bulk measurements [16,17]. Spatially resolved analyses have further demonstrated that transcriptionally distinct niche populations occupy characteristic anatomical locations and interact with specific hematopoietic compartments [18]. These advances provide a framework for examining radiation responses at multiple molecular levels. Transcriptomics can identify altered cellular states and regulatory programs; proteomics and secretomics can characterize functional signaling and intercellular communication; metabolomics can capture changes in energy, amino acid, and redox metabolism; and lipidomics can provide complementary information on membrane composition, lipid signaling, and oxidative lipid remodeling [15,16,17,18]. Integration of these molecular layers with spatially resolved profiling and absorbed-dose information is particularly relevant to RPT, in which radiation exposure can vary considerably across microscopic regions of tumor, bone, and marrow.

This review, therefore, examines radiation-induced remodeling of bone metastases and the bone marrow microenvironment across external-beam radiotherapy and radiopharmaceutical therapy. We first describe the anatomical and cellular organization of the bone–tumor–marrow ecosystem and the radiosensitivity of its hematopoietic and non-hematopoietic components. We then consider how EBRT and RPT perturb this eco-system through differences in spatial energy deposition, dose rate, exposure duration, particle range, and target localization. Particular attention is given to bone remodeling, oxidative and metabolic stress, immune and vascular responses, lipid remodeling, and hematopoietic injury. Finally, we discuss how transcriptomic, proteomic, metabolomic, lipidomic, and spatially resolved approaches can be integrated with dosimetry to distinguish biological changes associated with effective tumor control from those contributing to treatment resistance, skeletal dysfunction, and marrow toxicity.

In interpreting the literature, we distinguish among (i) direct clinical or tissue-based evidence from patients with bone metastases, (ii) findings from bone marrow, skeletal-cell, or metastatic-bone experimental models, and (iii) mechanistic evidence extrapolated from other radiation systems. Where direct evidence from bone metastases is unavailable, conclusions are presented as biologically plausible hypotheses rather than established clinical mechanisms.

The literature for this narrative review was identified through PubMed searches updated through October 2026. Search concepts included combinations of terms related to bone metastasis, bone marrow niche, radiotherapy, external-beam radiotherapy, radiopharmaceutical therapy, ^223^Ra, PSMA-targeted radioligand therapy, hematopoietic toxicity, oxidative stress, and transcriptomic, proteomic, metabolomic, lipidomic, single-cell, and spatial analyses. Priority was given to human clinical studies and direct evidence from bone metastases when available, followed by relevant bone marrow, skeletal, and other experimental radiation models used to provide mechanistic context. Because this was a narrative review, no formal meta-analysis or risk-of-bias assessment was performed.

## 2. Biology of the Bone–Tumor–Marrow Ecosystem

### 2.1. Anatomical Organization and Cellular Heterogeneity of Marrow Niches

The bone marrow is a spatially heterogeneous organ in which hematopoiesis, skeletal remodeling, vascular trafficking, and immune regulation occur within closely interconnected anatomical compartments. Early in vivo studies established bone-associated osteoblastic cells as regulators of hematopoietic stem cell (HSC) number and function [19,20]. Subsequent genetic and imaging studies shifted the field toward a distributed-niche model in which HSCs are supported predominantly by perivascular stromal and endothelial populations that provide stem cell factor (SCF) and CXCL12 [21,22,23,24]. Arteriolar, sinusoidal, and bone-associated regions are not mutually exclusive “niches” but overlapping microenvironments whose relative contribution changes with cell state, age, injury, inflammation, and malignancy [25,26,27,28,29].

The vascular compartment is itself functionally heterogeneous. Less-permeable arterial vessels are associated with lower reactive oxygen species (ROS) and HSC quiescence, whereas sinusoidal vessels permit hematopoietic-cell trafficking and are surrounded by abundant CXCL12-rich/LepR-positive stromal cells [25,28,29]. Type-H-like bone vessels additionally couple angiogenesis to osteogenesis [27]. These observations are relevant to metastasis because disseminated cancer cells encounter different oxygen tensions, stromal signals, nutrient conditions, and hematopoietic populations according to their anatomical location. Thus, the pretreatment substrate of a bone metastasis is not a uniform marrow cavity but a mosaic of microenvironments with different baseline biological functions.

### 2.2. HSPC Regulation by Stromal, Vascular, Immune, and Neural Cells

Hematopoietic stem and progenitor cells (HSPCs) do not function autonomously. Their quiescence, self-renewal, lineage commitment, retention, mobilization, and regeneration are controlled by signals from multiple cell populations. Perivascular mesenchymal stromal populations provide SCF and CXCL12, endothelial cells contribute trophic and trafficking signals, and osteolineage cells regulate bone-associated hematopoietic compartments [21,22,23,24]. Importantly, single-cell analyses have shown that conventional labels such as “mesenchymal stromal cell” or “osteoblast-lineage cell” comprise multiple transcriptionally and functionally distinct subpopulations [16,17,18,30]. This heterogeneity is relevant to radiation injury because cells that are anatomically close to one another may differ markedly in cell cycle status, antioxidant capacity, differentiation state, and hematopoietic-support function.

The niche also contains regulatory hematopoietic and neural elements. CD169-positive macrophages help retain HSPCs by maintaining stromal CXCL12-related programs [31], regulatory T cells can confer local immune privilege [32], and megakaryocytes directly regulate HSC quiescence and post-injury regeneration through CXCL4- and TGF-β-related signaling [33,34]. Marrow adipocytes provide a further context-dependent component; although excess adiposity is often associated with impaired hematopoiesis, adipocyte-derived SCF can support regeneration after myeloablative injury [35]. Sympathetic and circadian inputs also regulate HSPC trafficking [36]. Collectively, these findings support a network model in which normal hematopoiesis depends on interactions among stromal, vascular, immune, hematopoietic, adipocytic, and neural populations rather than on a single “master” niche cell.

### 2.3. Bone Remodeling and Establishment of the Metastatic Niche

The hematopoietic system is embedded within a continuously remodeling skeletal tissue. Osteoblast-lineage cells generate and mineralize bone matrix, osteoclasts resorb mineralized tissue, and osteocytes, stromal cells, marrow adipocytes, vessels, and extracellular-matrix components coordinate skeletal homeostasis [1,2,3,4,5]. Bone metastasis exploits this physiological remodeling machinery. Tumor-derived signals can alter osteoblast and osteoclast activity, while growth factors released or activated during bone turnover feed back to support tumor survival and expansion. In prostate cancer, overt lesions are commonly osteoblastic or mixed, but enhanced bone formation does not imply absence of osteoclastic activity; dysregulated formation and resorption coexist and contribute to the metastatic phenotype [5].

Metastatic colonization can also appropriate regulatory pathways normally used by hematopoietic cells. Prostate cancer cells have been shown to occupy HSC-supporting regions and compete for niche access [9]. The metastatic state is dynamic rather than fixed. Differentiated osteoblasts can impose quiescence on disseminated prostate cancer cells through TGF-β/GDF-related signaling [37], whereas remodeling of the endosteal niche can release dormant malignant cells in experimental bone disease [38]. Current work aims at identifying tumor-intrinsic programs that maintain bone-marrow dormancy and prevent metastatic outgrowth [39]. These observations are important for radiotherapy because the biological substrate exposed at treatment may range from a relatively intact marrow containing dormant disseminated cells to a heavily remodeled lesion in which tumor, bone, stroma, vessels, and normal hematopoiesis have already been reorganized.

### 2.4. Immune, Vascular, and Metabolic Features of Bone Metastasis

Bone metastases develop within an immune environment that differs from both uninvolved marrow and many soft-tissue tumor sites. In human prostate cancer bone metastases, single-cell analyses have identified tumor-associated macrophages, inflammatory monocytes, exhausted T-cell states, and actionable immunoregulatory signaling, illustrating substantial heterogeneity among immune and stromal compartments [40]. Such local immunosuppression provides one explanation for why systemic immune activation does not necessarily translate into equivalent antitumor immunity within bone.

**Figure 1:**
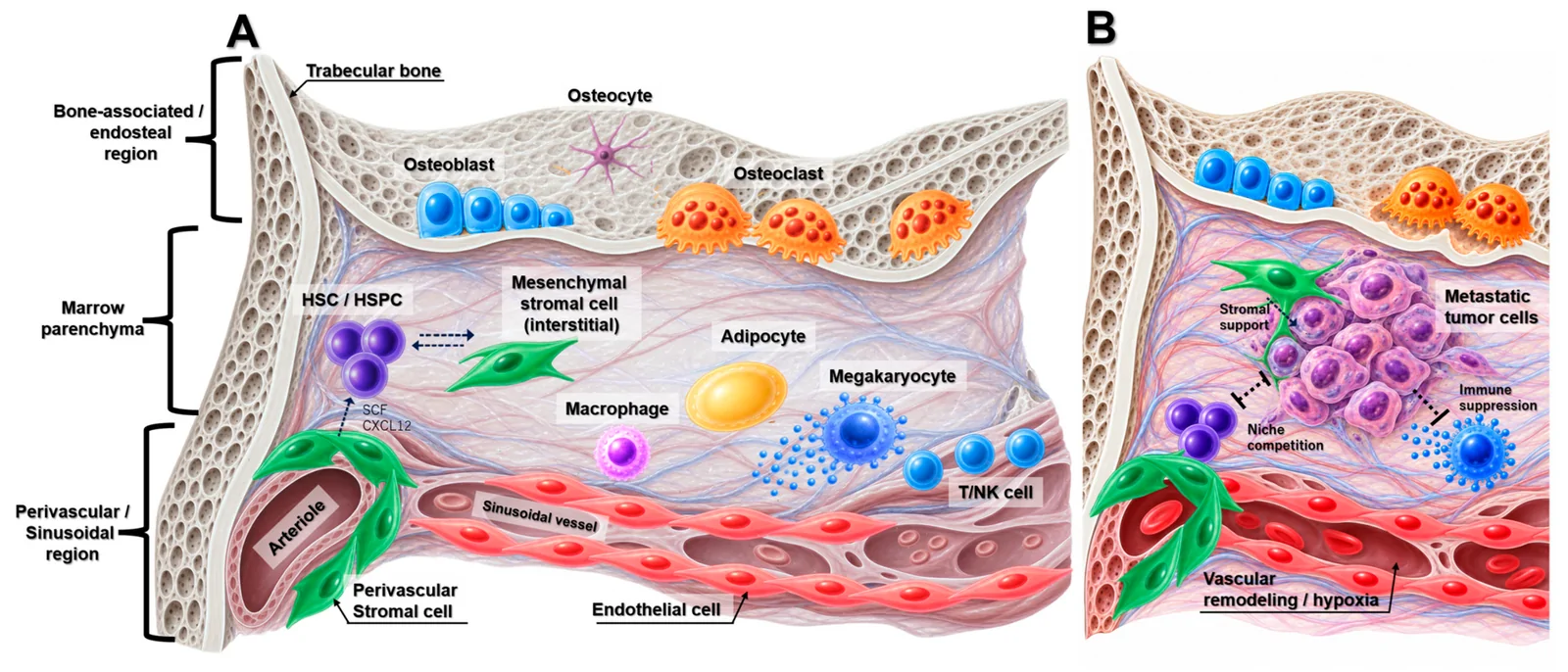
Anatomical and cellular organization of the bone–tumor–marrow microenvironment. (**A**) The normal bone marrow microenvironment comprises interconnected skeletal, hematopoietic, stromal, vascular, and immune compartments. Osteoblasts, osteoclasts, and osteocytes contribute to skeletal homeostasis, whereas mesenchymal and perivascular stromal cells, endothelial cells, macrophages, megakaryocytes, and other niche components support HSC/HSPC maintenance and trafficking, including through factors such as SCF and CXCL12. (**B**) Following metastatic colonization, tumor cells remodel these pre-existing compartments through stromal support, competition with hematopoietic niches, immune suppression, and vascular and hypoxic remodeling, while also perturbing bone remodeling. These interactions collectively establish a dynamic bone metastatic niche that can influence both tumor behavior and normal marrow function. In both panels, arrows (→) indicate supportive or promoting interactions, whereas blunt-ended lines (⊣) indicate inhibitory or competitive interactions.

Vascular architecture and metabolism are tightly linked to these immune and stromal states. Bone vessels regulate oxygen delivery, nutrient availability, tumor-cell trafficking, and hematopoietic-cell movement while remaining coupled to osteogenesis [27,29]. Expanding metastatic lesions can distort perfusion, intensify nutrient competition, and generate spatially heterogeneous hypoxia. Lactate accumulation, amino-acid availability, redox stress, and lipid use can then alter both tumor-cell fitness and immune-cell function. Therefore, before radiation is applied, a bone metastasis already represents a spatially heterogeneous tumor–bone–marrow microenvironment. This pre-existing heterogeneity is the biological substrate upon which different radiation modalities subsequently act. The anatomical and cellular organization of the normal and metastatic bone–tumor–marrow microenvironments is summarized schematically in Figure 1. The major cellular compartments of this microenvironment and their physiological, metastatic, and radiation-related roles are summarized in Table 1.

## 3. Radiation Perturbation of the Bone–Tumor–Marrow Microenvironment

### 3.1. Differential Radiosensitivity and Early Injury Across Hematopoietic and Niche Compartments

Ionizing radiation perturbs the bone–tumor–marrow microenvironment through direct energy deposition and secondary chemical damage, but the initial response differs substantially among cell populations. HSPCs are heterogeneous in radiosensitivity according to differentiation stage, cell-cycle status, surface phenotype, DNA-repair capacity, and antioxidant defenses [14,41,42]. Studies using human CD34-positive cells have demonstrated inter-individual differences in clonogenic survival and associations between immature phenotypes, Tie-2 expression, antioxidant-gene expression, and radiation response [41,42]. Megakaryocytic differentiation and platelet production provide an additional example in which progenitor proliferation is highly radiation sensitive, whereas terminal maturation of surviving cells may remain partly preserved [43,44]. High-LET exposure can alter these relationships further [45,46], and transcriptomic profiling has identified additional radiation-responsive programs in human HSPCs [47].

The non-hematopoietic niche also responds to radiation. Mesenchymal stromal cells, osteoblast-lineage cells, endothelial cells, macrophages, and other supporting populations may survive doses that strongly deplete hematopoietic progenitors yet undergo durable changes in differentiation, mitochondrial function, secretory phenotype, extracellular-matrix regulation, or hematopoietic support [15]. Consequently, marrow injury should not be interpreted solely as direct killing of hematopoietic cells. The functional consequences of the same physical dose depend on which compartments occupy the irradiated volume and on whether the marrow is intact, previously treated, or already replaced by metastatic disease.

### 3.2. Photon External-Beam Radiotherapy: Clinical Delivery, Fractionation, and Marrow Exposure

Photon EBRT remains the standard local treatment for symptomatic bone metastases. Contemporary ASTRO and ESTRO guidance supports several conventional palliative schedules, including 8 Gy in a single fraction and multifraction regimens such as 20 Gy in five fractions or 30 Gy in 10 fractions, with stereotactic approaches selected according to clinical context [10,48,49]. These guidelines are primarily designed around pain relief, neurological compromise, fracture risk, local control, and normal-organ constraints; they do not establish a universal bone-marrow dose constraint for focal treatment of bone metastasis.

Nevertheless, marrow exposure is biologically relevant. A vertebral or pelvic target can encompass substantially more active hematopoietic marrow than a lesion in an appendicular bone. In pelvic chemoradiotherapy, dose-volume measures of pelvic bone and functional marrow correlate with hematologic toxicity, and marrow-sparing planning can reduce cytopenias [50,51,52]. These data cannot be transferred directly to focal palliative bone-metastasis radiotherapy, because patient populations, concurrent chemotherapy, field size, and target geometry differ, but they establish a clinically important principle: the amount and functional status of marrow receiving a low-to-intermediate dose matter. For bone metastases, the planning target volume therefore represents only one layer of the biological problem; the composition and reserve of the irradiated skeletal region also influence the outcome.

### 3.3. Proton and Other External Particle-Beam Approaches: Selected Applications

Photon EBRT dominates routine treatment of bone metastases, whereas proton therapy and other external charged-particle approaches remain selected rather than standard options. Their principal physical advantage is the potential to reduce integral dose outside the target through the depth-dose characteristics of charged particles (the Bragg peak). This can be attractive for selected spinal lesions, reirradiation, or situations in which adjacent organs and marrow reserve are important. Recent retrospective series have reported feasibility and local control with hypo-fractionated or ablative proton therapy for spine metastases, including reirradiation settings [53,54]. However, clinical evidence remains limited compared with photon EBRT, and the effect of proton treatment on bone-marrow niche biology has not been systematically characterized. Carbon-ion therapy has an established role in selected radioresistant primary bone and soft-tissue tumors in some regions, but it is not a routine modality for metastatic bone palliation. Accordingly, external particle therapy is considered here as an important bridge between conventional photon EBRT and internally delivered particle irradiation, but it is not a major focus of this review.

### 3.4. Beta-Emitting Radiopharmaceutical Therapy: Prolonged Irradiation and Cross-Fire

Beta-emitting RPT differs fundamentally from EBRT because absorbed dose is determined by biodistribution, target expression, perfusion, uptake, retention, and physical decay. In contemporary prostate cancer practice, ^177^Lu-PSMA radioligand therapy is the major example [12,55,56]. Beta particles have a tissue range that permits cross-fire beyond the directly targeted cell. This can partially compensate for heterogeneous target expression but also exposes adjacent stromal, vascular, skeletal, and marrow compartments. Dose is delivered over hours to days rather than within minutes, and the dose rate decreases continuously as activity clears and decays.

These features complicate direct comparison with EBRT. The same lesion-averaged absorbed dose can correspond to different temporal patterns of DNA damage and repair, cell-cycle redistribution, and non-targeted effects. In addition, PSMA expression and radioligand uptake vary among lesions and within individual lesions. Marrow exposure is influenced by systemic activity, circulating radiopharmaceutical, skeletal disease burden, and loss of normal marrow reserve, and should be interpreted within established marrow dosimetry frameworks [57]. Bone tumor volume measured on PSMA PET has recently been associated with severe hematologic toxicity during ^177^Lu-PSMA therapy, illustrating the interaction between disease distribution and treatment-related marrow risk [58].

### 3.5. Alpha-Emitting Radiopharmaceutical Therapy: High-LET Microdosimetry and Target Localization

Alpha-emitting RPT generates a different pattern of microscopic energy deposition. Alpha particles have high linear energy transfer (LET) and short tissue ranges, producing densely ionizing tracks and complex damage. In bone-dominant mCRPC, ^223^Ra is particularly efficient because it localizes close to mineralized surfaces in areas of active bone turnover rather than requiring expression of a tumor-cell antigen [11]. Direct measurements in patient bone-metastasis samples have demonstrated marked small-scale heterogeneity, with the highest ^223^Ra-derived absorbed doses concentrated near the bone–tissue interface [59]. Thus, for ^223^Ra, the spatial relation among mineralized bone, osteoblast/osteoclast activity, tumor cells, and adjacent marrow is central to biological effect.

Investigational tumor-targeted alpha radioligands such as ^225^Ac-PSMA constitute a different form of alpha exposure [60,61,62]. Both ^223^Ra and ^225^Ac produce high-LET particles, but one is guided primarily by bone mineral turnover and the other by molecular tumor targeting. The identity of the initially irradiated cell populations and the microscopic dose distribution therefore differ. This distinction supports a key principle of this review: radiation quality alone is insufficient to predict biological response without also considering target localization and microanatomy.

### 3.6. Comparative Framework: From Macroscopic Dose to Cellular Exposure

**Figure 2:**
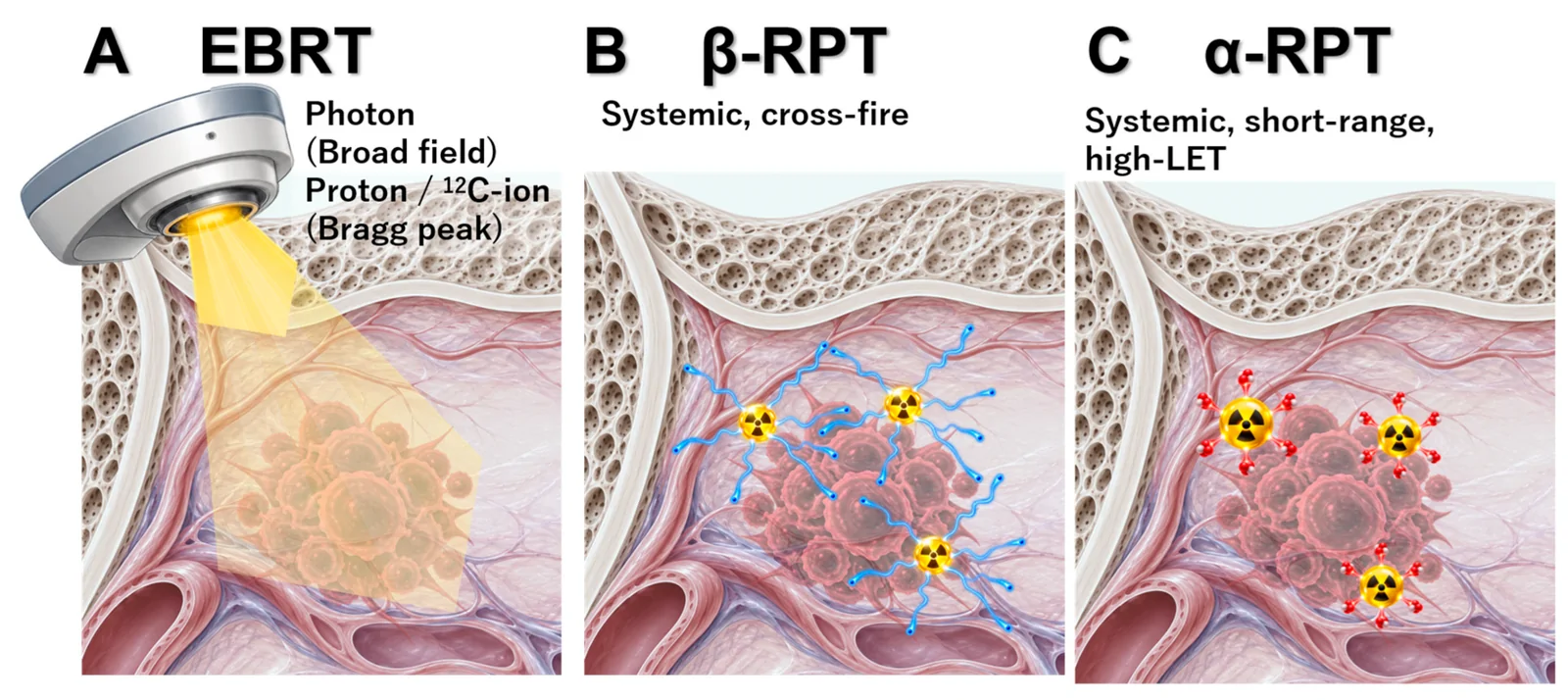
Schematic comparison of radiation-delivery patterns in the bone–tumor–marrow microenvironment. (**A**) EBRT delivers radiation from an external source across a relatively broad treatment field that may encompass the metastatic lesion and adjacent marrow. Proton and carbon-ion therapies are also external-beam modalities and are characterized by Bragg-peak dose deposition, although their radiobiological properties differ from those of photons. (**B**) β-RPT produces systemic, target-dependent irradiation with a comparatively longer particle range, enabling cross-fire irradiation of neighboring tumor and microenvironmental cells. (**C**) α-RPT produces highly localized, short-range irradiation with high LET and high biological effectiveness. The spatial distribution of RPT depends strongly on radionuclide localization and target expression; the schematic illustrates representative tumor-associated irradiation patterns. The figure is conceptual and not drawn to scale.

The most useful comparison among photon EBRT, external charged-particle therapy, beta-emitting RPT, and alpha-emitting RPT is multidimensional rather than a simple ranking of biological effectiveness. Relevant variables include LET, range, dose rate, exposure duration, microscopic dose heterogeneity, target localization, cross-fire, fractionation, and the amount of functional marrow within or adjacent to the exposed region. Photon EBRT provides externally controlled macroscopic geometry and brief, fractionated exposures; proton therapy modifies external depth-dose distribution while remaining a planned local treatment; beta-RPT combines molecular targeting with cross-fire and protracted dose delivery; and alpha-RPT produces highly localized high-LET tracks whose biological effect depends strongly on radionuclide microdistribution. These distinct radiation-delivery patterns in the bone–tumor–marrow microenvironment are illustrated schematically in Figure 2. Their major physical, biological, and clinical characteristics are summarized in Table 2.

A major knowledge gap is the scale mismatch between clinical dosimetry and biology. Treatment planning and RPT dosimetry commonly summarize exposure at the level of an organ, lesion, or voxel, whereas hematopoietic recovery, stromal remodeling, and tumor resistance emerge from interactions among individual cells and spatially organized niches. Bridging this gap requires microdosimetry and spatial biology, which are considered later in this review.

## 4. Biological Mechanisms of Radiation-Induced Niche Remodeling

### 4.1. Skeletal-Cell and Stromal Remodeling

Radiation can alter the balance between bone formation and resorption by affecting osteoblast-lineage cells, osteoclasts, osteocytes, and their stromal precursors. The direction and duration of these effects depend on dose, fractionation, baseline bone turnover, disease burden, and treatment modality. In metastatic bone, changes in osteoblast differentiation or osteoclast activity can modify both skeletal integrity and tumor-supportive signaling because bone remodeling regulates the local availability of growth factors and cytokines.

Recent alpha-RPT data provides a direct example of this principle. In experimental models, ^223^Ra caused trabecular bone loss, impaired osteoblast activity, a transient increase followed by suppression of osteoclast activity, and later marrow adipogenesis; zoledronic acid prevented the associated trabecular bone loss [66]. These findings support the concept that radiation effects on bone are not limited to tumor cells or mineralized matrix but involve coordinated responses among multiple stromal compartments. Bone-protective drugs are discussed as therapeutic interventions in Section 7; here, their importance is that they demonstrate the biological relevance of the irradiated skeletal niche.

### 4.2. Hematopoietic Dysfunction, Regeneration, and Long-Term Niche Memory

Acute marrow injury reflects depletion of radiosensitive progenitors and impaired production of mature blood cells, but recovery depends on more than HSC survival. Residual stem cells must re-enter the cell cycle, expand, differentiate, and receive appropriate support from vascular, stromal, immune, and metabolic components of the niche. Radiation-induced dysfunction in these supporting compartments can delay or qualitatively alter regeneration even when HSCs remain viable [14,15].

Long-term impairment can arise through persistent DNA damage, oxidative stress, HSC senescence, altered clonal composition, stromal remodeling, and vascular injury. The importance of redox control for HSC self-renewal is supported by experimental studies of ATM, p38 MAPK, and FOXO signaling [67,68,69]. In patients with extensive skeletal metastases, these mechanisms operate on a background of pre-existing replacement of normal hematopoietic space and cumulative systemic treatment. Therefore, a normal peripheral blood count at one time point does not necessarily indicate restoration of marrow reserve or normalization of all local niches.

### 4.3. Oxidative Stress, Mitochondrial Adaptation, and Antioxidant Defenses

Radiation generates ROS both through water radiolysis and through subsequent mitochondrial and enzymatic processes. In HSCs, excessive ROS can promote loss of quiescence, differentiation, senescence, and exhaustion; seminal studies established ATM-dependent control of oxidative stress, ROS–p38 MAPK signaling, and FOXO-mediated antioxidant regulation as important determinants of HSC maintenance [67,68,69]. These mechanisms provide a broader framework than any single antioxidant pathway.

NRF2-dependent transcription is one component of this network. Human HSPC studies have linked expression of NRF2-regulated genes with individual radiation responses [42], and irradiated osteoblastic/stromal models show NRF2-related oxidative-stress responses [15]. Surviving tumor and stromal cells can also adapt through glutathione metabolism, mitochondrial quality control, NADPH-generating pathways, and other antioxidant systems. Thus, redox adaptation has compartment-specific consequences: it can preserve normal HSPCs while simultaneously contributing to tumor or stromal radioresistance. This duality is central to evaluating redox-targeted radiosensitization strategies.

### 4.4. Metabolic and Lipid Remodeling

Radiation-induced adaptation extends beyond classical antioxidant defense. Glycolysis, mitochondrial respiration, amino-acid use, nucleotide metabolism, and nutrient competition can change after irradiation and thereby influence tumor survival, stromal support, and immune-cell activity. These effects are particularly heterogeneous in bone marrow because quiescent HSCs, cycling progenitors, stromal populations, immune cells, and tumor cells occupy distinct metabolic states.

Lipid metabolism provides a mechanistic bridge among membrane integrity, signaling, oxidative stress, and regulated cell death. Radiation can increase lipid peroxidation and contribute to ferroptotic cell death, while GPX4, glutathione, ACSL4-dependent phospholipid remodeling, and related lipid-defense pathways modify sensitivity [70,71,72,73]. Sphingolipids, including ceramides and sphingosine-1-phosphate, provide an additional signaling layer linking stress, survival, inflammation, and intercellular communication. In irradiated bone-marrow stromal and osteoblastic models, changes in phosphatidylcholines, lyso-phosphatidylcholines, sphingomyelins, and secreted metabolites have already been observed [15,74]. In this section these findings are interpreted as mechanisms of niche remodeling; the analytical role of lipidomics and metabolomics is separated into Section 5.

### 4.5. Inflammatory, Immune, and Intercellular Communication

Radiation can stimulate inflammatory and innate immune pathways through release of damage-associated signals, cytosolic nucleic acids, and stress-induced secretory programs. A well-characterized example is cGAS–STING/type-I interferon signaling, which can promote antigen presentation and antitumor immunity, but whose magnitude depends on radiation dose and DNA clearance pathways such as TREX1 [75]. At the same time, NF-κB signaling, IL-6, TNF, TGF-β, myeloid recruitment, and tissue-repair programs can support chronic inflammation, fibrosis, or immunosuppression. The net effect, therefore, depends on dose, fractionation, timing, baseline immune state, and the spatial relation between irradiated tumor and immune cells.

Communication extends beyond soluble cytokines. Extracellular vesicles, secreted proteins, metabolites, lipid mediators, and bystander signals can transmit radiation responses to cells that received little or no direct radiation dose. Experimental studies have implicated exosomes in radiation-induced bystander signaling [76]. This concept is especially relevant to marrow, where tumor, stromal, vascular, immune, and hematopoietic compartments are separated by short distances. It provides a biological explanation for why remodeling may extend beyond the highest-dose microregions.

### 4.6. Vascular and Hypoxic Remodeling

Vascular injury can alter perfusion, permeability, endothelial signaling, and leukocyte trafficking. These changes may improve or worsen local oxygenation depending on timing and on subsequent vascular repair. Hypoxia is a classical modifier of low-LET radiation sensitivity because oxygen promotes fixation of radiation-induced damage, but its biological importance extends far beyond this radiochemical effect. HIF-dependent programs regulate metabolism, stem-like states, angiogenesis, extracellular-matrix remodeling, and immune suppression [77,78,79].

Normal marrow itself contains spatial oxygen gradients and functionally distinct vascular territories [26,29]. Consequently, radiation-induced vascular damage can alter both tumor oxygenation and environmental cues that regulate HSPCs. For RPT, heterogeneous uptake and prolonged exposure may create temporal patterns of vascular and hypoxic adaptation that differ from those produced by a brief photon fraction. Direct comparative data in bone metastases are still limited, making paired spatial dosimetry and vascular/hypoxia mapping an important future research direction.

## 5. Systems-Level Characterization of the Irradiated Bone–Marrow Microenvironment

### 5.1. Transcriptomics and Single-Cell Transcriptomics

Transcriptomic profiling can identify radiation-induced changes in DNA-damage responses, inflammatory signaling, lineage differentiation, stress adaptation, and cell-state transitions. Bulk RNA analysis remains useful when tissue quantity is limited, but it averages signals across the highly heterogeneous cellular composition of marrow and bone metastases. Single-cell RNA sequencing can separate tumor, stromal, endothelial, immune, and hematopoietic populations and can identify rare or transitional states that may be obscured in bulk data [16,17,18,30].

Longitudinal single-cell analysis is particularly attractive for separating transient radiation responses from persistent remodeling. Recent single-cell studies of irradiated hematopoietic systems have begun to identify radiation-resistant HSC subsets and niche-to-HSPC signaling programs [80]. However, dissociation bias and loss of anatomical context remain major limitations, motivating integration with spatial approaches.

### 5.2. Proteomics and Secretomics

Proteomics provides information closer to functional cellular machinery than transcript abundance alone. Radiation can alter protein abundance, phosphorylation, mitochondrial proteins, extracellular-matrix components, antioxidant enzymes, and signaling networks without proportional changes in mRNA. Secretomics adds a complementary dimension by characterizing cytokines, growth factors, extracellular proteins, and other molecules released into the local environment.

This is particularly relevant in marrow because niche function depends strongly on cell–cell communication. In irradiated BMSCs and osteoblast-lineage cells, proteomic changes in oxidative-stress and mitochondrial pathways coexist with changes in secreted lipids [15]. Such molecular changes suggest that surviving niche cells may undergo persistent functional remodeling; however, proteomic or secretomic alterations alone do not establish impaired hematopoietic support or enhanced tumor-supporting function, which requires independent functional validation.

### 5.3. Metabolomics

Metabolomics captures the biochemical phenotype that emerges from gene and protein regulation. It can resolve changes in glycolytic intermediates, tricarboxylic-acid-cycle metabolites, amino acids, nucleotides, redox couples, and other small molecules that reflect energy demand, oxidative stress, and biosynthetic activity. In contrast to transcriptomics, metabolomics can reveal rapid pathway-level consequences of radiation before stable transcriptional remodeling is established.

Its main limitation in complex tissue is source attribution. A metabolite detected in marrow, plasma, or conditioned medium may arise from tumor, stromal, hematopoietic, immune, or vascular cells. Cell-defined models, isotope tracing, paired intracellular/extracellular analysis, and integration with transcriptomic or proteomic data can improve interpretation. Alpha-irradiated osteoblastic-cell models already demonstrate the feasibility of identifying radiation-associated changes in secreted metabolites and candidate signaling pathways [74].

### 5.4. Lipidomics

Lipidomics can be viewed as a specialized metabolic layer that resolves membrane composition, lipid signaling, energy storage, and oxidative lipid damage at a depth not captured by routine metabolomic panels. Relevant lipid classes include phosphatidylcholines, lyso-phosphatidylcholines, sphingomyelins, ceramides, sphingosine-1-phosphate, fatty acids, and oxidized phospholipids. These readouts connect directly to the mechanisms discussed in Section 4.4, including membrane remodeling, lipid peroxidation, ferroptosis, and sphingolipid signaling.

For this review, lipidomics is not presented primarily as a circulating-biomarker platform. Its value is as a tool for understanding local bone/marrow biology. Secreted lipid changes after irradiation of BMSCs and osteoblastic cells provide proof of concept that niche-cell lipid output is radiation responsive [15]. Future studies should determine whether different modalities—photon EBRT, beta-RPT, and alpha-RPT—produce distinct lipid states in defined marrow compartments.

### 5.5. Spatial Multi-Omics and Integration with Dosimetry

The major limitation of conventional omics is loss of spatial information. This is particularly problematic in radiation biology, where physical dose delivery is inherently spatial. Spatial transcriptomics, multiplex imaging, spatial proteomics, imaging mass spectrometry, and other approaches can assign molecular states to anatomical regions and specific cell populations. A recent in silico study combining spatial transcriptomics with pharmacokinetic and dosimetric modeling of ^177^Lu- and ^225^Ac-based RPT illustrates how spatial target expression, hypoxia, cell composition, and predicted absorbed dose can be integrated in prostate cancer [81].

**Figure 3:**
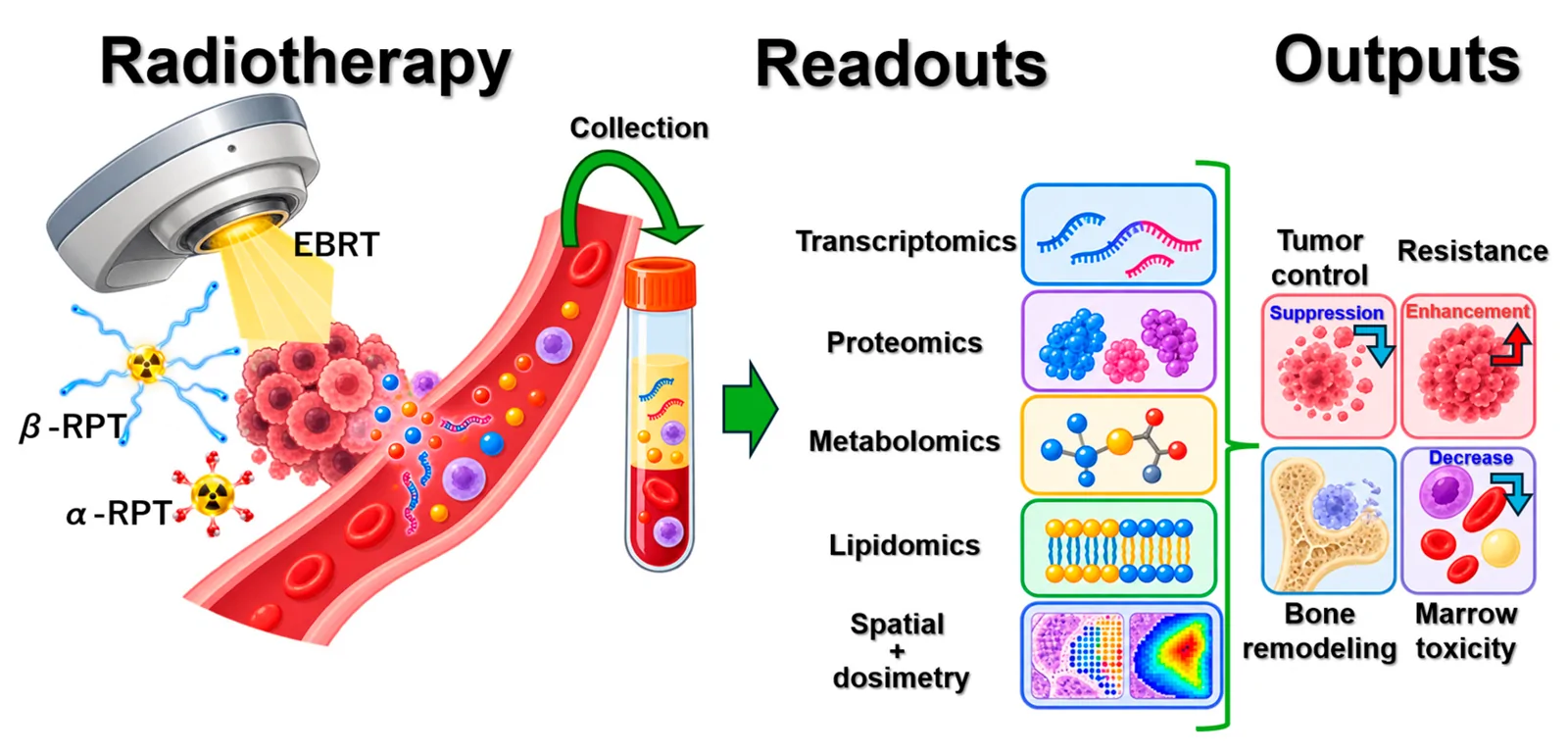
Integration of molecular readouts, spatial profiling, and dosimetry to characterize radiation-induced remodeling of the bone–tumor–marrow microenvironment. EBRT and RPT perturb both metastatic tumor cells and the surrounding bone marrow microenvironment, generating molecular changes that can be assessed in circulating and tissue-derived samples. Transcriptomic, proteomic/secretomic, metabolomic, and lipidomic analyses provide complementary molecular readouts, while spatial profiling and dosimetry define where these biological changes occur and how they relate to local radiation exposure. Integration of these information layers may help connect heterogeneous radiation responses with clinically relevant outcomes, including tumor control, treatment resistance, bone remodeling, and marrow toxicity. The figure is schematic and not drawn to scale.

The next step is to apply similar approaches directly to bone metastases and marrow. For EBRT, planned dose maps can be registered with spatial tissue data; for RPT, quantitative activity imaging, pharmacokinetic modeling, and microdosimetry are required. Integrated multi-omics and spatially resolved approaches may, therefore, be needed to decipher how distinct radiation qualities and dose-delivery patterns reshape the bone–tumor–marrow microenvironment. Linking these molecular profiles with spatial dosimetry may help distinguish therapeutic remodeling from changes associated with resistance and marrow toxicity. The integration of radiation exposure, molecular and spatial readouts, and clinically relevant outcomes is summarized schematically in Figure 3.

## 6. Clinical Consequences: From Tumor Control to Marrow Toxicity

### 6.1. Tumor Response, Dormancy, Resistance, and Recurrence

Clinical response in bone metastases is determined not only by intrinsic tumor-cell radiosensitivity but also by target expression, perfusion, stromal support, hypoxia, immune state, and treatment-induced adaptation. This is especially apparent in RPT, where different lesions in the same patient may receive different absorbed doses. Persistent or dormant tumor cells may subsequently exploit a remodeled niche to survive or regrow. Spatial heterogeneity in target expression and dose delivery therefore provides a plausible biological link between lesion-level response variability and eventual recurrence [81].

### 6.2. Skeletal Consequences of Treatment

Changes in osteoblasts, osteoclasts, matrix, and vasculature can affect bone turnover, structural strength, fracture risk, pain, and repair. The clinical importance of skeletal complications and bone protection was highlighted by findings from ERA 223 and the PEACE-3 safety analysis, in which the use of bone-protecting agents was associated with substantially lower fracture incidence [63,64]. However, these clinical observations do not establish that fractures result solely from direct radiation-induced injury to the bone microenvironment, because systemic treatment, baseline skeletal risk, and trial design may also contribute. Separately, preclinical studies provide mechanistic evidence that ^223^Ra can affect trabecular bone, osteoblast activity, osteoclast dynamics, and marrow adipogenesis [66]. Together, these findings support the clinical relevance of skeletal remodeling while emphasizing that the mechanisms underlying treatment-associated fractures are likely multifactorial.

### 6.3. Hematologic Toxicity and Marrow Recovery

Hematologic toxicity reflects an interaction among absorbed dose, active marrow volume, baseline reserve, prior therapies, disease burden, and replacement of normal hematopoietic tissue by tumor. In ^177^Lu-PSMA therapy, larger PET-defined bone tumor volume has been associated with severe hematologic toxicity [58]. In small clinical study of patients receiving ^223^Ra, urinary 8-hydroxy-2′-deoxyguanosine (8-OHdG), a non-invasive marker of oxidative DNA damage, was associated with subsequent bone marrow suppression [65]. Although urinary 8-OHdG has been investigated as a radiation-response biomarker in other irradiation settings, its predictive value for hematologic toxicity during RPT remains to be validated in larger prospective cohorts.

### 6.4. Clinical Integration in Bone-Dominant Metastatic Prostate Cancer

Although the bone–tumor–marrow framework described here is intended to capture biological principles that may be relevant across skeletal metastases, much of the current clinical evidence for radiopharmaceutical therapy discussed in this review is derived from prostate cancer, particularly studies of ^223^Ra and PSMA-targeted therapies. Extrapolation to bone metastases from other malignancies should therefore be made cautiously, because the balance of osteoblastic and osteoclastic remodeling, immune and stromal context, molecular targets, and available treatment options can differ substantially among tumor types.

Bone-dominant mCRPC is a useful clinical model because several radiation modalities can act on different compartments during the same disease course. EBRT is used for focal symptomatic or oligo-progressive lesions; ^223^Ra localizes to areas of active bone turnover; ^177^Lu-PSMA targets PSMA-expressing tumor; whereas investigational ^225^Ac-PSMA approaches are being evaluated as high-LET tumor-targeted irradiation strategies. Preclinical studies indicate that α- and β-emitting RPT can produce distinct immunomodulatory effects [82], while recent PET and immuno-histologic data suggest that ^223^Ra can modify PD-L1-associated features within bone metastases [83]. This modality-specific biological heterogeneity supports careful consideration of treatment sequence and marrow reserve in patients who may receive multiple radiation-based therapies.

## 7. Therapeutic and Translational Implications

### 7.1. Matching Modality, Dose, and Sequence to Disease Distribution and Marrow Reserve

Treatment selection should consider not only tumor burden but also the spatial relationship between disease and functional marrow. EBRT provides precise local dose control, whereas RPT treats multiple sites systemically but produces uptake-dependent marrow exposure. Future planning frameworks could integrate prior radiation fields, active-marrow imaging, lesion distribution, baseline blood counts, and RPT dosimetry. The goal is not to minimize marrow dose at the expense of tumor control, but to identify where marrow reserve is most vulnerable and where dose can be redistributed without compromising efficacy.

### 7.2. Combination with Systemic Anticancer Therapy

Androgen-receptor pathway inhibitors, chemotherapy, PARP inhibitors, and immune therapies can modify both tumor radiosensitivity and marrow reserve. Combination strategies therefore require evaluation of hematologic and skeletal outcomes in addition to tumor response. The ERA 223 and PEACE-3 studies underscore the need to consider multiple factors when evaluating the skeletal safety of combination treatment, including treatment context, concomitant systemic therapy, baseline fracture risk, and appropriate use of bone-protecting agents [63,64,84]. Combination of ^177^Lu-PSMA with pembrolizumab has also shown clinical activity, providing a basis for further investigation of RPT–immunotherapy sequencing [85].

### 7.3. Targeting Bone and Stromal Compartments

Bone-modifying agents already demonstrate that the metastatic microenvironment can be therapeutically targeted. Denosumab and zoledronic acid are established options for reducing skeletal complications in mCRPC with bone metastases [86], and the PEACE-3 safety analysis showed that mandatory bone-protecting therapy substantially reduced fracture risk in the ^223^Ra setting [64]. Future niche-directed approaches may include modulation of osteoblast–osteoclast coupling, TGF-β signaling, vascular support, or tumor–stroma communication. Because these pathways also support normal repair and hematopoiesis, efficacy should be assessed together with marrow recovery and skeletal integrity rather than as tumor endpoints alone.

### 7.4. Targeting Redox and Metabolic Vulnerabilities

Redox and ferroptosis pathways are attractive radiosensitization targets because radiation itself generates oxidative and lipid stress. However, normal HSPCs and niche cells also depend on tightly regulated ROS and glutathione homeostasis [67,68,69]. The therapeutic objective is therefore selective exploitation of tumor metabolic vulnerabilities rather than indiscriminate amplification of oxidative damage. At present, NRF2-, GSH-, GPX4-, ACSL4-, and ferroptosis-directed strategies should be presented as mechanistic and preclinical opportunities rather than established clinical recommendations.

### 7.5. Toward Individualized Tumor–Marrow Treatment Planning

A useful long-term goal is to move from a tumor-dose-only perspective toward a three-axis framework integrating (i) tumor dose and target coverage, (ii) marrow dose and functional reserve, and (iii) local niche state. Multi-omics is unlikely to be required routinely for every patient; rather, discovery studies can identify imaging or blood-based surrogates that capture clinically useful features of the local microenvironment. Such a framework could support treatment decisions that maximize tumor control while preserving enough functional marrow for subsequent systemic and radiation therapies. For clinical translation, transcriptomic and proteomic signatures may help identify inflammatory, stromal, or regenerative states associated with treatment response or delayed marrow recovery, whereas metabolomic and lipidomic profiles may reveal candidate markers of oxidative and metabolic stress. These findings could subsequently be translated into practical blood-, urine-, or imaging-based biomarkers and prospectively validated against clinically relevant endpoints, including lesion response, significant cytopenia, hematologic recovery, and skeletal complications.

## 8. Future Perspectives

Future studies should connect radiation physics directly with the cellular and spatial biology of bone and marrow. A practical prospective study could enroll patients before EBRT or RPT and obtain baseline imaging, complete blood counts, measures of marrow reserve, and blood and urine samples, followed by treatment-specific dosimetry and predefined longitudinal sampling during treatment and hematologic recovery. When clinically feasible, paired tumor or marrow samples could provide complementary spatial and cellular information. Single-cell and spatial approaches could then identify which cellular states emerge in high-dose versus low-dose microregions and which are associated with durable control, resistance, fracture, or hematologic toxicity. Relevant clinical endpoints would include lesion-level response, clinically significant hematologic toxicity, time to blood-count recovery, skeletal complications, and subsequent treatment tolerance. Multi-omics and spatial analyses could initially be performed in discovery cohorts, with candidate imaging or circulating biomarkers subsequently tested in independent validation cohorts.

A central challenge is that EBRT, beta-RPT, and alpha-RPT do not share a simple one-to-one dosimetric scale. Comparative studies should therefore map modality-specific exposure variables onto common biological outcomes rather than assuming equivalence from mean absorbed dose alone. Spatial transcriptomics coupled with modeled RPT dosimetry already provides a proof of concept for this direction [81]. Artificial intelligence and integrative modeling may further help combine imaging, dosimetry, and multi-omics, but models should remain interpretable and require prospective validation before clinical use.

The ultimate objective is precision treatment of the bone–tumor–marrow microenvironment: selecting and sequencing radiation modalities according to disease distribution, target expression, skeletal biology, marrow reserve, and the capacity of the surrounding niche to recover after treatment.

## 9. Conclusions

Bone metastases should not be regarded simply as tumor deposits within mineralized tissue. They develop within a dynamic bone–tumor–marrow microenvironment in which malignant cells interact with skeletal, stromal, vascular, immune, and hematopoietic compartments. External-beam radiotherapy and RPT perturb this system through distinct spatial and temporal patterns of energy deposition, and their biological effects extend beyond direct tumor-cell killing. Radiation-induced changes in bone remodeling, hematopoiesis, redox and metabolic homeostasis, immune signaling, and stromal and vascular function can contribute both to therapeutic efficacy and to treatment resistance, skeletal dysfunction, and marrow toxicity.

Future treatment strategies should therefore move beyond consideration of tumor dose alone. Integration of tumor and marrow spatially resolved dosimetry with complementary transcriptomic, proteomic, metabolomic, and lipidomic information may provide a more comprehensive framework for understanding individual radiation responses. Such approaches could ultimately help distinguish beneficial antitumor remodeling from injury to the supporting bone marrow environment and enable more individualized use of external-beam radiotherapy and radiopharmaceutical therapy.

## Figures and Tables

**Table 1:** Major cellular compartments of the bone–tumor–marrow microenvironment.

Cell/Compartment	Typical Location	Core Physiological Role	Role in Bone Metastasis	Radiation Relevance
HSC/HSPC	Perivascular/sinusoidal and other marrow niches	Self-renewal; hematopoietic output	Niche competition/displacement; reduced reserve	Direct radiosensitivity; long-term effects depend on differentiation state and dose
Mesenchymal stromal cells (LepR+/CXCL12-rich subsets)	Perivascular marrow	SCF/CXCL12 support; osteogenic/adipogenic potential	Tumor-supportive signaling; matrix/metabolic remodeling	Secretory, metabolic and differentiation responses can alter hematopoietic support
Osteoblast-lineage cells	Bone surfaces/osteogenic regions	Bone formation; local niche regulation	Prominent interaction with prostate cancer; osteoblastic remodeling	Differentiation, redox, secretome and metabolic changes after irradiation
Osteoclasts	Bone-resorption surfaces	Bone resorption; coupling to bone formation	Matrix release; vicious cycle; dormancy/reactivation	Turnover changes after EBRT/RPT; relevant to skeletal toxicity
Osteocytes	Mineralized bone matrix	Mechanosensing; bone-remodeling signals	Potential regulator of metastatic environment	Exposed during EBRT; RPT effects less defined
Endothelial/perivascular cells	Sinusoidal and arteriolar compartments	Trafficking; oxygen/nutrient delivery; HSC support	Tumor homing; angiogenesis; hypoxia regulation	Vascular injury/permeability; altered niche support
Marrow adipocytes	Marrow cavity	Energy storage; context-dependent regenerative support	Metabolic signals to tumor/niche	Can expand after injury; may influence regeneration
Macrophage/myeloid cells	Marrow and metastatic niche	Immune surveillance; niche regulation	Immunosuppression, inflammation, tumor support	Polarization/inflammatory responses can be radiation-modulated
Megakaryocytes	Marrow, near vessels/HSC regions	Platelet production; HSC quiescence/regeneration	Indirect niche effects	Potential contributor to post-injury regeneration
T/NK cells and other lymphocytes	Marrow and metastatic lesions	Immune surveillance	Exhaustion/regulatory states suppress antitumor immunity	Activation or suppression depends on dose and modality
Metastatic tumor cells	Bone metastatic lesion/marrow niches	Not physiological	Colonization, dormancy, outgrowth, niche competition	Primary therapeutic target; response shaped by niche state and modality

**Table 2:** Comparison of radiation-delivery patterns relevant to bone metastases and marrow.

Feature	Photon EBRT	Proton/External Particles (Selected Use)	β-Emitting RPT	α-Emitting RPT
Representative examples	Conventional EBRT, hypofractionation, SBRT	Proton spine RT; selected reirradiation	^177^Lu-PSMA-617	^223^Ra; investigational ^225^Ac-PSMA
Delivery	Externally planned local field	Externally planned local field with charged-particle depth-dose	Systemic, uptake-dependent internal irradiation	Systemic, uptake-dependent internal irradiation
LET	Low	Low for proton; higher/variable for carbon ions	Low	High
Exposure duration	Minutes per fraction	Minutes per fraction	Hours–days, declining dose rate	Protracted radionuclide kinetics; dense local tracks
Spatial heterogeneity	Beam geometry and anatomy	Beam geometry, range and distal fall-off	Biodistribution, target expression, uptake/retention, cross-fire	Microscopic localization of activity; short range
Target localization	Anatomical target volume	Anatomical target volume	Usually molecular target expression	Bone-seeking (^223^Ra) or molecular tumor targeting (^225^Ac-PSMA)
Marrow exposure	Depends on field location/volume and active marrow included	Potential integral-dose sparing; clinical marrow data limited	Systemic/heterogeneous; influenced by disease burden and activity	Potentially focal near bone surfaces or targeted tumor; microdistribution critical
Key biological question	Which local compartments are encompassed by the field?	Can normal tissue/marrow sparing translate to biological benefit?	How do prolonged exposure and cross-fire alter tumor–niche–marrow balance?	How does high LET microdistribution affect tumor versus niche cells?
Major evidence gap	Direct spatial niche-response data after modern schedules	Limited use in metastases and limited marrow-biology data	Direct spatial mapping of marrow/niche responses	Human data linking microdosimetry to niche remodeling and hematopoietic injury
Representative clinical effects/evidence	Established for pain palliation and local control of symptomatic bone metastases; marrow effects depend on irradiated volume and location [10,48,49,50,51,52]	Feasible for selected spine metastases and reirradiation; comparative clinical marrow data remain limited [53,54]	^177^Lu-PSMA improves disease control and survival in selected PSMA-positive mCRPC; systemic marrow toxicity remains clinically relevant [12,55,56,58]	^223^Ra improves survival in bone-predominant mCRPC [11]; skeletal complications and marrow toxicity require consideration [63,64,65]; ^225^Ac-PSMA remains investigational [60,61,62]

## Data Availability

No new data were created or analyzed in this study. Data sharing is not applicable to this article.

## Abbreviations

EBRT	external-beam radiotherapy
RPT	radiopharmaceutical therapy
SBRT	stereotactic body radiotherapy
HSC	hematopoietic stem cell
HSPC	hematopoietic stem and progenitor cell
MSC	mesenchymal stromal cell
BMSC	bone marrow stromal cell
SCF	stem cell factor
CXCL12	C-X-C motif chemokine ligand 12
mCRPC	metastatic castration-resistant prostate cancer
PSMA	prostate-specific membrane antigen
LET	linear energy transfer
ROS	reactive oxygen species
NRF2	nuclear factor erythroid 2-related factor 2
GSH	glutathione
GPX4	glutathione peroxidase 4
cGAS	cyclic GMP–AMP synthase
STING	stimulator of interferon genes
IFN	interferon
NF-κB	nuclear factor κB
TGF-β	transforming growth factor β
EV	extracellular vesicle
TCA	tricarboxylic acid
PC	phosphatidylcholine
LPC	lysophosphatidylcholine
SM	sphingomyelin
S1P	sphingosine-1-phosphate
